# Model Calibration of Pharmacokinetic-Pharmacodynamic Lung Tumour Dynamics for Anticancer Therapies

**DOI:** 10.3390/jcm11041006

**Published:** 2022-02-15

**Authors:** Maria Ghita, Charlotte Billiet, Dana Copot, Dirk Verellen, Clara Mihaela Ionescu

**Affiliations:** 1 Research Group of Dynamical Systems and Control, Ghent University, 9052 Ghent, Belgium; maria.ghita@ugent.be (M.G.); dana.copot@ugent.be (D.C.); 2 Faculty of Medicine and Health Sciences, Antwerp University, 2610 Wilrijk, Belgium; 3 EEDT—Core Lab on Decision and Control, Flanders Make Consortium, 9052 Ghent, Belgium; 4 Cancer Research Institute Ghent, 9052 Ghent, Belgium; 5 Department of Radiation Oncology, Iridium Cancer Network—GZA Hospitals Sint Augustinus, 2610 Wilrijk, Belgium; charlotte.billiet@gza.be (C.B.); dirk.verellen@uantwerpen.be (D.V.); 6 Department of Radiotherapy, Faculty of Medicine and Health Sciences, Antwerp University, 2610 Wilrijk, Belgium; 7 Department of Automatic Control, Technical University of Cluj Napoca, 400114 Cluj, Romania

**Keywords:** lung cancer, tumor growth, mathematical model, treatment planning, patient response, optimal dosing therapy, pharmacokinetic-pharmacodynamic

## Abstract

Individual curves for tumor growth can be expressed as mathematical models. Herein we exploited a pharmacokinetic-pharmacodynamic (PKPD) model to accurately predict the lung growth curves when using data from a clinical study. Our analysis included 19 patients with non-small cell lung cancer treated with specific hypofractionated regimens, defined as stereotactic body radiation therapy (SBRT). The results exhibited the utility of the PKPD model for testing growth hypotheses of the lung tumor against clinical data. The model fitted the observed progression behavior of the lung tumors expressed by measuring the tumor volume of the patients before and after treatment from CT screening. The changes in dynamics were best captured by the parameter identified as the patients’ response to treatment. Median follow-up times for the tumor volume after SBRT were 126 days. These results have proven the use of mathematical modeling in preclinical anticancer investigations as a potential prognostic tool.

## 1. Introduction

Described as a societal challenge of the modern world [1], the lung cancer paradigm leads the research progress towards increasing the effectiveness of curative treatment options, with positive outcomes in decreased morbidity and improved patient life. The scientific community recognizes the difficulties encountered in lung cancer, proposing a shift of paradigm towards a multidisciplinary approach to reach drug dosing optimality [2,3,4,5].

The prognostic and predictive implications of biological response after treatment in terms of describing tumor dynamics are captured by mathematical models, validated with in vitro experimental or clinical data. In the context of tumor modeling, the classical approach provides [6,7]:

- exponential-linear models [8,9],
- logistic and Gompertz models [9,10,11],
- dynamic carrying capacity models [12],
- Von Bertalanffy and power law models [13,14].

Most principles of tumor growth are expressions of ordinary differential equations [15], but recent advances suggest that complex biological phenomena can be captured by adopting fractional calculus with non-integer order differentiation [16,17,18,19,20,21,22,23]. Quantitative analysis can be done in a variety of experimental settings, including in vitro and in vivo studies, involving animal or human data, for preclinical anticancer drug investigations in all existing therapies (i.e., radiotherapy, chemotherapy, immunotherapy, targeted drug therapy) [24,25,26,27,28]. Tumor growth kinetics follow the purely phenomenological approach from an initial exponential phase followed by a linear regimen, a mathematical formalism that can be characterized as pharmacokinetic and pharmacodynamic modeling [29,30,31].

In this work, a pharmacokinetic-pharmacodynamic (PKPD) model of radiotherapy is developed with a description of the following features: (i) the proliferation of the tumor, (ii) the necrosis of tumor cells, (iii) the clearance and inhibitory effect of the therapy, and (iv) the therapeutic effect of ablative radiation. The model is a revision of our previous work described in [29] and adjusted for integrating the rationale for dose fractionation, according to each patient RT schedule. For lung tumors, the computed dose distributions strongly depend on the accuracy of the predictions based on the model. In SBRT, a very high biologically effective dose (BED) is delivered to the center of the targeted tumor, while delivering a high dose to the clinical target volume and a minimized dose to normal tissue. Detailed theoretical analyses concluded that higher BED irradiated during a short period must be administrated to achieve successful local control of lung cancer cell dynamics.

The interventional study presented here provides one of the first investigations into how mathematical PKPD parameters correlates with SBRT dose distributions for patients with primary NSCLC or lung metastases. It has a pivotal role in using mathematical formulations in predicting the clinical outcome after SBRT in real treatment applied on patients, instead of in vitro studies or in vivo on animal models (i.e., mice). Our work provides an important opportunity to advance the understanding of using mathematical models as tools in treatment planning for describing the current state and evolution of lung tumours, depending on inter- and intra-patient variability. This realistic PKPD model allows for different time-dose schemes for therapy administration to be evaluated and compared, taking into account the diffusion pattern and tumor dynamics observed. Since radiation-induced pneumonitis is a severe side-effect whose probability to occur primarily depends on the total dose and the volume of irradiated tissue, the dose-calculation algorithm is considered highly relevant in treatment planning support. Prediction of tissue behavior concerning the model parameters can have differences in patient and tumor characteristics, length of time interval follow-up, and definition of requirements to validate the tumor progress. This model is then a cross-fertilization between current medical practice information tools and prediction models available for tumor growth.

We conducted a small pilot study of 22 patients with non-small cell lung cancer (NSCLC) treated with stereotactic body radiation therapy (SBRT) in order to analyze in mathematical terms the relationship between a given physical absorbed dose and the resulting biological response, quantifying the influencing factors as well. The obtained results for tumor volume are comparable to the measured variables in clinical practice, for the same radiotherapy (RT) regimen. Nevertheless, given the treatment management particularity established from histology, molecular pathology, age, comorbidities, and patient preferences, patient-to-patient variability in response to RT is still debatable for the influence on the development of individualized treatment modality.

The main scope of this work is to calibrate and validate the application of PKPD model for SBRT in patients with NSCLC and encourage its use in the decision-making process about treatment management, as a recommended additional supportive tool.

## 2. Materials and Methods

### 2.1. Clinical Study Design

The data set for this study was assessed under a non-randomized clinical trial performed at GasthuisZusters Hospital Sint-Augustinus in Antwerpen, Belgium, entitled “Respiratory impedance models for non-invasive lung function testing in individualized stereotactice body radiation therapy (RIMIRT)” (CTOR20105GZA). Prior to commencing the study, ethical clearance was provided. The study was approved by the Medical Ethics Committee GZA and was conducted in accordance with the Declaration of Helsinki, the guidelines for Good Clinical Practices (ICH-GCP), and any applicable regulatory requirements.

The results presented in this work provide a proof of concept for the proposed modeling strategy by means of calibrating the PKPD model with the tumor volume from the patient database. The procedures performed throughout the clinical study are outlined following the events schedule:

1. Screening visit (patient inclusion). Eligibility of the patient is determined according to the inclusion/exclusion criteria and screening assessments are recorded, as shown in Table 1 (i.e., anthropometric data, diagnosis, the extent of cancer, tumor characteristics and localization, medical history, concomitant medication, spirometric and plethysmographic data (if available)).
2. Intervention phase. Once enrolled, fully eligible patients will start with the intended SBRT treatment and specific clinical information about the scheme of treatment is provided (dose fractionation, dose distribution details, duration of administration, and concomitant therapies.) The tumor volume was measured by the radiation oncologist using CT scans done for RT treatment simulation.
3. Follow-up phase (usually 3 months after last treatment day). The tumor volume was measured by the radiation oncologist using CT scans done after RT administration, during the follow-up visit.

In this flow diagram, the use of the forced oscillation technique (FOT) as a maneuverless, easy to perform a lung function test is introduced. Details on the use of this technique are given in [16,19,32,33]. The complementary information from the FOT measurements is out of the scope of this paper, but the end goal of the clinical study is to minimize model uncertainty by injecting respiratory mechanics variables into the PKPD model.

**Table 1:** Characteristics of participants completing the study.

Characteristic	Value (*n* = 19 Patients)	%
Age (y)		
Median	67.84	(46–80)
Sex		
Men	12	63.2%
Women	7	36.8%
Site of original primary tumor		
Lung	10	52.6%
Colon	4	21%
Rectum	2	10.5%
Other sites ^1^	3	15.8%
Primary tumor histology		
Adenocarcinoma	9	47.4%
Spinocellular carcinoma	4	21%
Mucinous carcinoma	1	5.3%
Clear cell carcinoma	1	5.3%
Unknown/NA	4	21%
Primary or metastatic lung lesion		
Primary	9	47.4%
Metastatic	10	52.6%
TNM Classification of Malignant tumors ^2^		
T0N0M1	10	52.6%
T1N0M0	7	36.8%
T2N0M0	2	10.5%
Tumor localization (by lobe side)		
Right side	12	63.2%
Left side	5	26.3%
Both sides	2	10.5%
Tumor localization (by tumor position within the lobe)		
Upper lobe	7	36.8%
Lower lobe	9	47.4%
Upper and lower lobe	2	10.5%
Mid lobe	1	5.3%
Number of lesions		
1 lesion	16	84.2%
2 lesions	2	10.5%
3 lesions	1	5.3%
ECOG Performance Status ^3^		
ECOG 0	7	36.8%
ECOG 1	11	57.9%
ECOG 2	1	5.3%
Smoking history		
Active	9	47.4%
Ex-smoker	5	26.3%
Never	5	26.3%
Respiratory disorders		
COPD ^4^	8	42.1%
Other respiratory disorders ^5^	2	10.5%
NA	9	47.4%
Medical history		
Previous surgeries ^6^	8	42.1%
Previous RT ^7^	4	21%
No previous interventions	9	47.4%
Fractionation schemes		
1 × 34 Gy	2/22	9%
3 × 18 Gy	9/22	40.9%
4 × 12 Gy	10/22	45.5%
8 × 7.5 Gy	1/22	4.5%
Concomitant cancer therapy		
Chemotherapy—Xeloda (Capecitabine)	1	5.3%

*Abbreviations:* NA = not applicable, ECOG = Eastern Cooperative Oncology Group, COPD = Chronic Obstructive Pulmonary Disease, GOLD = Global Initiative for COPD. ^1^ Other sites: sigmoid, hypopharynx, kidney. ^2^ TNM is a globally recognised standard for classifying the anatomical extent of tumor cancers. TNM stage is classified at the time of RT treatment. T: size or direct extent of the primary tumor (T0: no evidence of tumor, T1, T2: size and/or extension of the primary tumor), N: degree of spread to regional lymph nodes (N0: no regional lymph nodes metastasis), M: presence of distant metastasis (M0: no distant metastasis, M1: metastasis to distant organs). ^3^ ECOG Performance Status: 0-Asymptomatic (fully active, able to carry on all predisease activities without restriction), 1-Symptomatic but completely ambulatory (restricted in strenuous activity, ambulatory and able to do light work.), 3-Symptomatic, <50% in bed during the day (capable of all self care, but no work activities, out of bed >50% of day). ^4^ COPD types: GOLD 3 (severe) and GOLD 4 (very severe). ^5^ Other respiratory disorders: asthma, pneumonitis. ^6^ Previous surgeries of the: lung cancer (pneumonectomy, lobectomy), kidney (nephrectomy), colon cancer, breast cancer. ^7^ Previous radiation treatment for: lung, prostate, hypopharynx.

### 2.2. Participants Data

**Figure 1:**
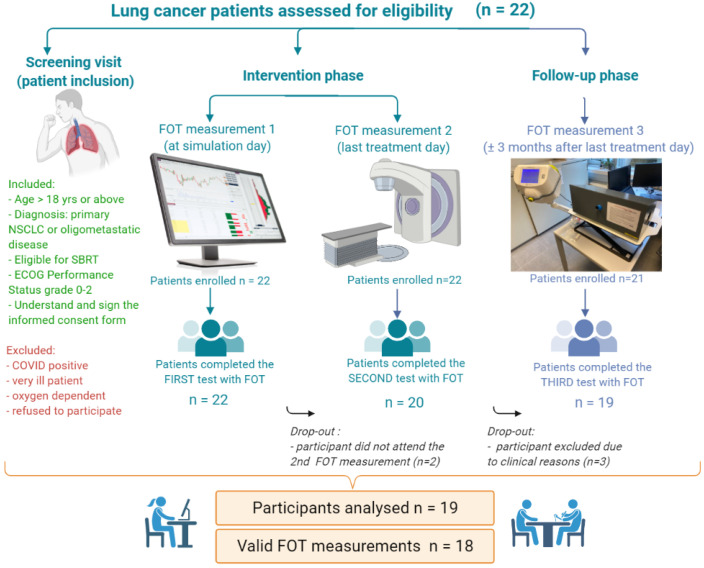
Flow diagram of the study design.

The study included a total of 22 patients (19 eligible) with NSCLC, who are medically inoperable or high-risk surgical candidates, treated with various combinations of dose-rate and fractionation. The detailed flow diagram of the study participants can be depicted in Figure 1. Advanced technologies were used for delivering curative RT safely, given by linear accelerators producing X-rays with high-energy [1,3,34,35].

The patients enrolled in the interventional pilot study satisfied all the inclusion criteria: male or female, aged 18 years or above, diagnosed with primary NSCLC or oligometastatic disease eligible for SBRT, having an Eastern Cooperative Oncology Group (ECOG) Performance Status grade 0–2. Each patient signed an informed consent form (ICF) indicating that he/she understands the purpose and procedures required for the study and is willing to participate in the study and comply with all the requirements.Three patients are missing from reported data, one did not attend the second and third visit of the study, while two patients were drop-out due to clinical reasons of disease progression.

The characteristics of the participants that completed the study are listed in Table 1. 19 patients were eligible, of which 3 patients had multiple lesions treated separately. Therefore, a total of 22 treatments schemes specific to each tumor were considered. Data collected during the screening evaluation includes, but is not limited to demography, diagnosis, and extent of cancer (site of original tumor, histology, primary or metastasis, TNM stage, tumor localization, number of lesions), ECOG performance status, smoking history, medical history (previous RT treatments and/or cancer resection surgeries, respiratory disorders), cancer resection surgeries), fractionation schemes and concomitant cancer therapy. At the follow-up visit, the patients underwent computed tomography (CT), for evaluation of treatment efficacy and the tumor volume is measured again (if still exist). For the purpose of comparison principles, the oncologist performed the same method of tumor measurement as used for the screening visit.

### 2.3. Treatment Strategy—Clinical Protocol

Of the various lung cancer treatment protocols, SBRT is the standard of care for medically inoperable patients with peripherally located, early-stage NSCLC (non-small cell lung cancer) or for the patients that refuse the surgical resection of the tumor.

Decisions about the recommended radiotherapy regimen were based on a multidisciplinary discussion to provide consensus for SBRT applicability, safety, and risks, in order to better assist management care and individualized treatment. For SBRT simulation, planning, and delivery, commonly used prescription doses and normal tissue dose constraints were taken into consideration by the medical team from GZA hospital, based on published experience and current guidelines [36,37,38,39,40], ongoing trials, historical data, modeling, and empirical judgment. Treatment planning was performed using a TrueBeam linear accelerator (Varian Medical Systems) and dose calculations were carried out with a colapsed cone convolution algorithm (RayStation TPS, RaySearch Laboratories) characterised by a set of user-definable configuration parameters [41,42].

The treatment planning uses prior knowledge highlighted in clinical practice recommendations for stereotactic treatments [36,37,38,39,40,43,44]. The dose and fractionation regimens, as presented in Table 2, are patient-specific and limited by a subset of historically used maximum dose constraints. However, the regimens used can be included in 4 subgroups, based on a multidisciplinary discussion, weighing the potential benefit over the patient risk.

Tumor size is determined by measuring the “tumor area” defined from the CT scans and is represented as a multiplication of the largest diameter of the tumor by the greatest perpendicular diameter [45]. The tumor volume is assessed twice: before RT (from the CT scan done for RT treatment simulation) and after RT (from the CT scan done after RT treatment during the follow-up visit, usually after 5 months). The time between the measurements is expressed for each patient, this criterion being important for the simulations.

For predicting the risk of radiation-induced toxicity (radiation pneumonitis (RP)) in future research, other dose-volume histogram (DVH) parameters are assessed. First, the lung V20 is defined as the percentage of normal lung receiving at least 20 Gy, being proportional with the total lung volume (TLV) [46]. Second, the mean lung dose (MLD) is expressed as the average dose of the CT-defined TLV [47]. Finally, lung V5 is introduced as a new dose constraint in treatment planning where TLV receiving a dose of 5 Gy should not exceed 60% [48].

### 2.4. Mathematical Formulation

Pharmacokinetic and pharmacodynamic interactions were simulated based on our prior work [29] which contained relevant model coefficient values from the literature. This revision is proposed by employing the PKPD compartmental model to real clinical data from patients with NSCLC. To extend the utility of the existing model, its parameters and structure were reviewed for analysing the clinical outcome obtained in clinical practice for the applied treatment, quantified by measuring on CT images the tumor area before and after SBRT.

Taking into consideration the proliferating tumor volume (*x*_1_ in mm^3^) and the necrotic tumor volume (*x*_2_ in mm^3^), the total tumor volume can be expressed. Since the exact therapy of each patient is simulated, it is not argumentative for the tumor volume to possess comparable dimensions with the ones reported by clinicians. In this model representation, the inhibitor level *x*_3_ in mg/(mL · day) is expressed based on the radiation dose rate administrated *u_r_* (mg · day/mL), according to the dosing regimen for NSCLC. The equivalent mass of the tumor from approximation formula 1 mm^3^ = 10^−3^ mg [34,49] was used to calibrate radiotherapy model parameters via a unit transformation from Gy ⟶ 1/mL.

Considering these features, the tumor growth dynamic model, entitled as PKPD model, has been characterized using the following equations:

(1) $$\begin{array}{cccc} {\dot{x}}_{1} & = & \left(a-n\right)x_{1} & -E\cdot x_{1}\\ {\dot{x}}_{2} & = & nx_{1} & +E\cdot x_{1}\\ {\dot{x}}_{3} & = & -c_{a}x_{3} & +u_{a}\\ {\dot{xe}}_{3} & = & -c_{a}xe_{3} & +Et_{a}\cdot x_{3}\\ {\dot{x}}_{4} & = & -c_{i}x_{4} & +u_{i}\\ {\dot{xe}}_{4} & = & -c_{i}xe_{4} & +Et_{i}\cdot x_{4}\\ {\dot{x}}_{5} & = & -c_{r}x_{5} & +u_{r}\\ {\dot{xe}}_{5} & = & -c_{r}xe_{5} & +Et_{r}\cdot x_{5} \end{array}$$

where *a* denotes the tumor growth rate, *n* the necrosis rate, and $c_{r}$ is the clearance rate on the Michaelis-Menten kinetics $\frac{x_{1}x_{3}}{ED50_{r}\;+\;x_{3}}$ (mm${}^{3}$/day). The parameters $xe_{i}$ and $Et_{i}$ are the effect drug concentrations, and the synergic effect between tumor cells and chosen therapy [50]. The model is a representation of combined therapies, respectively antiangiogenic, immunotherapy, and radiotherapy, respectively. Combining the effects of radiation therapy with the tumor incidence, produces a dose-response relationship, with a Hill slope deciding the interaction factor:

(2) $$I=Un_{t}+Un_{r}+\sigma Un_{r}\cdot Un_{t}$$

with σ denoting the amount of synergy present between the drugs. As a function of concentration, the Hill equation is expressed through the effect drug concentrations $xe_{i}$ which is normalized to its corresponding half effect concentration $C_{50}$.

(3) $$Un_{r}=\frac{U_{r}}{C_{50r}}$$

To solve the pharmacology of the therapy used, the Hill equation is considered as follows:

(4) $$Effect=\frac{I^{\gamma }}{1+I^{\gamma }}$$

where γ describes the patient drug responsiveness or drug resistance (curve’s sigmoidicity generated while using the Hill equation).

Table 3 provides the parametric model coefficients for the generic tumor representation. Given the GTV tumor volume provided by CT screening (from Table 2), this can be used to calibrate the PKPD model. The equivalent mass of the tumor from approximation formula 1 mm^3^ = 10^−3^ mg [29] was used to calibrate radiotherapy model parameters via a unit transformation from Gy ⟶ 1/mL. In order to set a reference value for the tumor value, we propose to quantify the volumetric difference between total tumor tissue and necrotic tissue, that is the effective volume still active in the tumor (in mm^3^):

(5) δVt=TotalVolume−NecroticVolume

Given the fact that in clinical practice for our study case, no other therapies have been used for the presented patients, the PKPD model coefficients that correspond to immunotheraphy and antiangiogenesis therapy, respectively, have been set with 0 values for model calibration. The proposed model can be further investigated with the purpose of evaluation of the synergic effects caused by the use of multiple anticancer therapies.

**Table 3:** Coefficients parameters corresponding with PKPD model applied on the study cohort.

Parameter	Name	Value	Units	Source
*a*	tumor growth rate	0.693	1/day	[34,49]
*n*	necrosis rate	0.10	1/day	[34,49]
$c_{r}$	clearance rate RT	3/treatment days	1/day	[34]
$C50_{r}$	half-effect concentration RT	20	Gy/day	[34]
$C50_{t}$	half-effect tumor growth	50	% mm^3^	[34,51]
$Emax_{r}$	max effect RT	50	%	[34,51]
γ	patient response	varies (0.043-0.25)	(-)	[52]
σ	drug reaction (synergic)	8	(-)	[52]
*E*	combined effects (all)	calculated	1/day	NA
$u_{r}$	radiotherapy dose rate	varies	mg/(mL·day)	Table 2

*Abbreviations:* NA = source not available.

### 2.5. Analysis

In order to examine the model sensitivity to unknown variability of the tumor volume, an analysis was made. Specifically, tumor volume uncertainty has been introduced and the end result in tumor volume decrease has been examined. Although the CT scans are delivering accurate information on tumor volume, it is not intended as a recurrent tool of investigation for patient safety and minimizing radiation risks. It is, therefore, necessary to allow uncertainty in the model while providing the medical staff with a certain measure of error they can accommodate in their tumor dynamics predictions.

The tumor volume reported using CT images was used as the baseline for comparison with the simulated values obtained with the PKPD model. A linear approach for modeling the relationship between the two sets of values for tumor volume after SBRT was used, as well as the display of all tumor measurements corresponding to each patient. As different dose regimens of therapy were administrated, their efficacy was separately evaluated.

To analyse the treatment efficacy, the percentage change in tumor volume (*TV*) was calculated using the following formula:

(6) $$Efficacy\left(\%\right)=100-\left(\frac{TV_{final}}{TV_{initial}}\right)\cdot 100$$

where $TV_{final}$ describes the tumor volume measured from the second CT scan in the last day of patient monitoring, as depicted in Table 2, and $TV_{initial}$ is the tumor volume acquired in the first CT scan on the simulation day.

## 3. Results

### 3.1. Prediction of Tumor Volume

**Figure 2:**
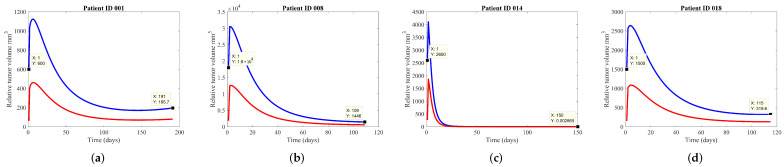
Representations of the PKPD model fitting the clinical data of the tumor volume for the study cohort, after SBRT treatment. The blue line represents the total tumor volume, while the red line is the necrotic tumor volume. On each figure, it is also represented the initial tumor volume and final tumor volume with their corresponding day, specific to each patient. Plotted are the tumor growths for the following patients: (**a**) patient ID 001, (**b**) patient ID 008, (**c**) patient ID 014, (**d**) patient ID 018.

The main rationale that guided our representation of tumor growth is the argument that the tumor existent at the lung site is represented by the total tumor volume (i.e., ‘alive’ and ‘dead’ tumor cells), and the washout of the dead tumor cells that suffered from apoptosis is still in process. Therefore, the measured volume for CT screening of the patients is taking into account the entire tumorous mass. In Figure 2, examples of the data for the patients with single lesions treated with SBRT are reported, having subsequently similar volumes with the ones measured and detailed in Table 2. In the plotted analysis it can be observed that the PKPD model fitted the same growth curve, corresponding with the *a priori* estimate of measurement.

We succeeded in closely matching the clinical data by varying the γ parameter related to patient response to treatment and keeping σ at the constant value of 8. The reasoning for keeping σ constant is explained by the use of a single therapy, instead of multiple concomitant therapies, therefore the synergy between treatments applied does not exist and is considered only the effect of radiotherapy in tumor cells. Parameter values resulting from the fits showed that increased γ (with values from 0.1 to 0.25) allows the tumor volume to decrease and achieve values close to 0. This can signify in clinical practice that the tumor has been eliminated as reported and is not measurable. Decreasing γ< 0.1, the model fits the tumor with a decreased curve of growth, but still existent. However, the parameters depend also on the maximal initial tumor volume. For the cases where the tumor volume is rather stable after therapy (no decrease), γ has also the smallest value (0.043), the model allowing the changes in the curvature. Those three patients where this situation is encountered (as mentioned in Table 2) are going to be further under investigations, as follow-up CT scans highlighted evident radiation pneumonitis (RP) reaction, leading to a discrete higher volume which is difficult to differentiate from the nodule.

It can be observed that the cellular radiosensitivity is not influenced by the size of the irradiated volume, rather on the RT dosing and intensity, although the consequence of cell death for lung is strongly dependent on initial volume.

### 3.2. Tumor Growth for Multiple Lesions

**Figure 3:**
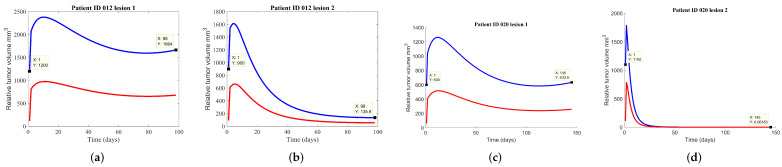
Representations of the PKPD model fitting the clinical data of the tumor volume for the patients with multiple lesions, after SBRT treatment. The blue line represents the total tumor volume, while the red line is the necrotic tumor volume. Each figure also represents the initial tumor volume and final tumor volume with their corresponding day, specific to each patient. Plotted are the tumor growth dynamics for the patients with multiple lesions: (**a**,**b**) patient ID 012, (**c**,**d**) patient ID 020.

Tumors with multiple lesions in the same site (lungs) have been identified before treatment. Having multiple lesions requires an adapted treatment that will treat separately each tumor. Given the diagnosis, treatment of multiple lung nodules is guided by the same regulations as the normal tumor. Accordingly, those lesions were treated by the clinicians separately, as single sites, without influencing each other. The results have shown that in clinical practice the multiple lesions are difficult to minimize, in the majority of the cases the lesions still remaining active with the same volume after treatment, as seen in Figure 3.

### 3.3. Uncertainty Analysis

The continuing uncertainty in tissue properties and tumor volume is of particular interest for high-precision delivery techniques for completing radiotherapy treatments in one or a few fractions.

**Figure 4:**
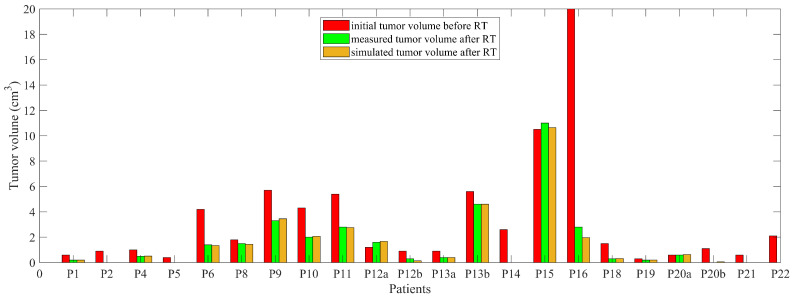
Tumor volumes reported for the included patients. It can be seen that there are patients with tumor eradication due to treatment, having tumor volume values close to 0. Two lesions (a and b) were analysed for patients 12, 13, and 20.

Modeling the changes in tumor dynamics seems to play an important role in modulating clinical responses. Notably, the representation of the tumor volumes reported for the included patients illustrates the close similarity between the simulated tumor volume and measured one after radiotherapy for all patients (Figure 4).

**Figure 5:**
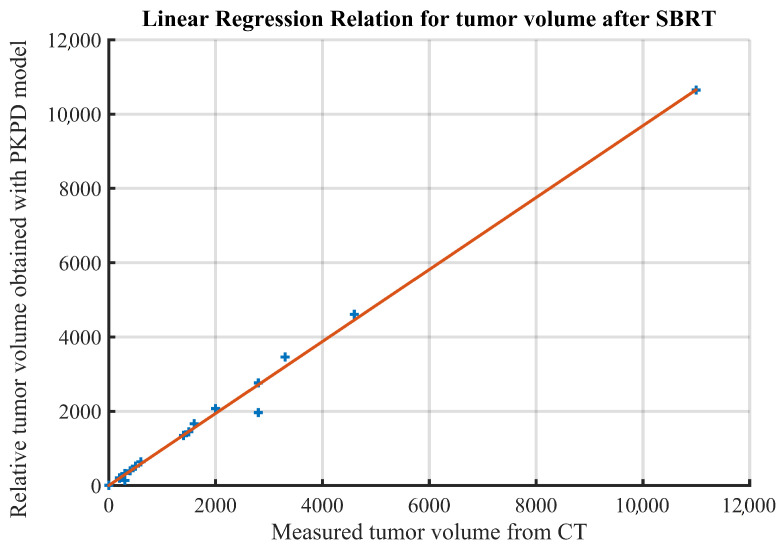
Linear regression showing strong correlation between the two volume measurements: measured from CT image and simulated with PKPD model.

We have analysed the error between the tumor volume after treatment reported by clinicians and the one obtained through model simulations, plotting the results. In Figure 5, the clinical reported volume is plotted against the relative simulated volume, together with the regression line (root mean squared error and correlation coefficient *R* = 0.994, and slope of the regression = 0.968). As expected, the two measures are strongly correlated.

**Figure 6:**
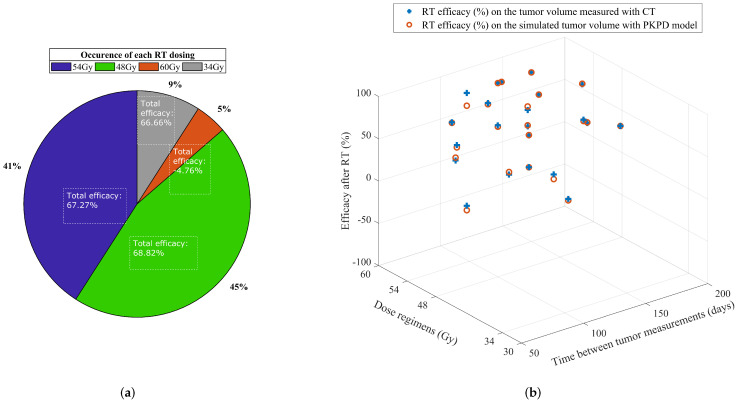
Occurrence of each fractionation regimen and their efficacy percentage for each patient. (**a**) Occurence of the fractionation regimens used for the study cohort. (**b**) Regression and progression in tumor volume after radiotherapy per patient, according to the calculated efficacy, dose regimen and time duration between measurements.

Further investigation of the effectiveness of each SBRT fractionated regimen in NSCLC patients shows a higher rate for 48 Gy/4 fractions (68.82 %), 54 Gy/3 fractions (67.27 %), and 34 Gy/1 fraction (66.66 %), compared to 60 Gy/8 fractions (−4.76 %) (as seen in Figure 6a). As illustrated in Figure 6b, depending on the patient response and fractionation regimen, the initial tumor volume has remained stable after treatment for 3 lesions, and decreased for all the other lesions (efficacy between 17.86% and 100%).

**Figure 7:**
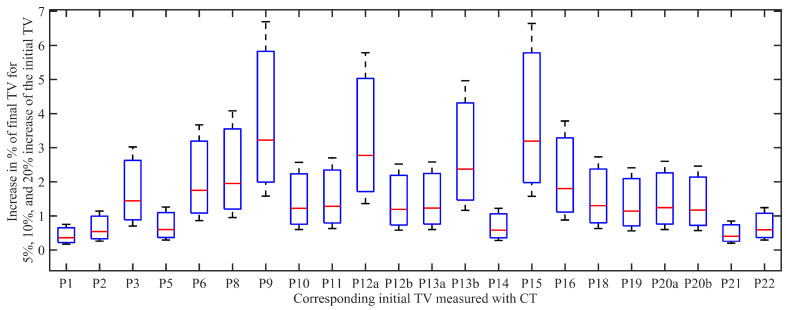
Box plot corresponding to changes in % for the tumor volume of each patient. On each box, the central mark indicates the median, and the bottom and top edges of the box indicate the 25th and 75th percentiles, respectively. The minimum score for each box is for the case of 5% increase in the initial tumor volume, while the maximum score is corresponding to 20%.

In the context of different methods for tumor measurements, we have simulated with the PKPD model the influence of the measurement errors that could occur in the tumor volume. With the scope of analysing the model uncertainty, we have decreased the initial tumor volume with 5%, 10%, and 20%, respectively (see Figure 7). Patients without a regression in the tumor met the biggest increased variation in the simulated final tumor volume (1.57–6.69%), concomitant with the increase in the initial tumor volume. However, for a change of the initial tumor volume, the final tumor volume after RT changes below 1% (for 5% decrease in *TV*), 0.5–3.22% (for 10%), and 0.85–6.69% (for 20%).

## 4. Discussion

Mostly, SBRT is frequently applied as hypofractionation with few large fractions higher than 2 Gy [34]. The therapeutic advantage is that it will increase in tolerance for the late-responding normal tissue damage than for tumors compared with conventional fractionation. In practice, SBRT uses image guidance and a coordinate system to locate the tumor in the lungs in order to treat it accurately. However, an interdisciplinary approach for individualized SBRT is recommended to achieve better dose conformity. Different time-dose schemes for therapy administration are evaluated, taking into account the tumor dynamics and synergy pattern observed from the PKPD model.

Our approach was to implement a mathematical formulation for describing the pharmacokinetics and pharmacodynamics of the tumor growth process under influence of radiation. The recommended dosages administrated were as follows: 54 Gy or 48 Gy for lesions situated mostly in the peripheral areas, without organs at risk nearby, a new scheme of a very high dose of 34 Gy in only 1 fraction (similar with 54 Gy) for very small tumor located peripherally and 60 Gy for centrally located lesions. The higher doses are radiobiological similar to all the other dosages, but the difference is the strength and the number of fractions. Therefore, centrally located lesions that are close to the trachea, bronchi, heart and are sensitive to high RT receive lower multiple doses because of the serious damage that can be caused to the healthy surrounding tissue. In our study, we had one patient with a resistant clone group of tumor cells not really sensitive to RT, so the patient receives systemic chemotherapy with Xeloda drug daily every two weeks.

Mathematical models describe through relatively simple laws the internal interconnecting complexity of tumor growth kinetics and dynamics. For further clinical exploration, a quantitative analysis of the tumor growth characterized particularly by the PKPD model was performed. In our analysis, the PKPD model was able to accurately fit both data sets, consistently with the reported values before and after the radiotherapy. As expected, our results confirmed previous observations that tumor growth is patient dependent in the range of the tumor volumes studied, validating the fractionation regimen used. However, the magnitude of local pulmonary changes is independent of the irradiated volume but does depend on patient-related factors such as co-morbidities, smoking, and age.

All the presented results emphasise a steep increase in tumor volume after the first applied dose of radiation. It can be explained as a local adverse effect that occurs in the tissue due to inflammation caused by the radiation. This first reaction is compensated in time, after further administration of radiotherapy.

Despite generic waveform similarities, important differences were noted in the parameter γ estimates for changes in the curvature of the tumor growth. This explains the fact that each patient has a different response for the treatment applied, i.e., patient variability. Our results and methodology provide a complementary aiding tool to predict the possible treatment outcomes. Despite the small dataset, the results are still representative in terms of the feasibility of clinical relevance.

In reviewing the literature, limited data was found on the association between input parameters from pharmacokinetic and pharmacodynamic models and SBRT in human NSCLC studies [53]. Most of the prior studies report in vitro data from animals studies, results used to project potential response in vivo. Comparison of the findings with those of other studies can only be done for the clinical outcome of SBRT in clinical trials.

**Figure 8:**
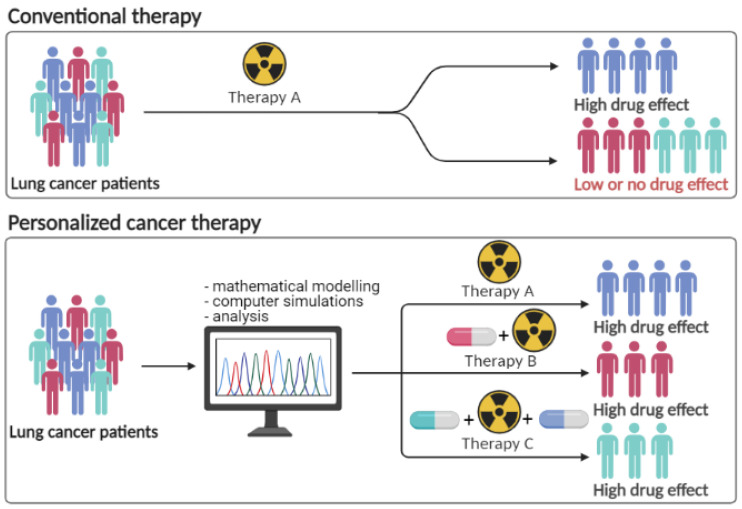
Precision Cancer Therapy versus Conventional Therapy.

Furthermore, this analysis is significant in the rational design of dose and scheduling of anticancer drugs for integration of therapies effects on tumor growth. By matching pre- and post-treatment measurements, the results show that the response of tumors to the same fractionation schemes is variable and we can also conclude that such heterogeneity also exists between multiple lesions of the same organ in patients. Although treatment scheduling is done by a multidisciplinary team, the same modified fractionation schedule may or may not be optimal for all patients. To best select the specific group to which a patient belongs is critical for maximizing benefits and can be changed in relevant preclinical settings, and therefore to individualize treatment (Figure 8).

In this study, we showed that high radiation doses lead to improved local tumor control, with tolerated toxicity. Usually, a decrease in the tumor size after RT can be seen in the time interval from weeks to two months, not immediately, all patients remaining with a visible local scar after radiation. We found that tumor volume GTV measured after RT decreased (86.36%) or stayed stable (13.63%) for all lesions. After further investigations of the CT scans for the patients that preliminary results showed a greater field of unhealthy tissue after treatment, we distinguished between tumor and toxicity reaction. Via the examined scans, it was reported no tumor increase, closer inspection revealing a field of toxicity that can be graded with clinical factors and symptoms of RP, but possible to diagnose only after 6 months–1 year. Because of the presence of adjacent pneumonitis or fibrosis, the interpretation of the nodule area measurement is more difficult.

After diagnosis and staging lung cancer, a multidisciplinary team of health professionals proposes several comprehensive treatment schemes for each patient, mostly based on empirical knowledge, experience, and prior expertise from literature. The multidisciplinary oncologic consult (MOC) could involve these mathematical predictions when a patient’s case is discussed while taking into consideration the standard imaging tests, laboratory results, and clinical condition of the patient. The selection of the best treatment option in the pretreatment scheduling phase is benefiting from the computer simulations of growth dynamical models of treatment response. The detection of different patterns of response in the chosen therapy according to specific responses criteria is bringing several advantages. These are as follows: help in designing interventions for cancer control initiatives, developing non-invasive techniques for precised therapies, reduced clinical trials, health gain in case of early treatment, life years gained, complications avoided.

Our approach explicitly offers an evidence on the calibration of the PKPD model with real patients’ data. The scope of this mathematical formalism is to provide a prediction of treatment effectiveness in patients scheduled for SBRT treatment strategies in NSCLC cohorts and, where possible, the extent of toxicity resulting from it. Therefore, this methodology assist the physicians and healthcare professionals involved in making treatment decisions.

Choosing SBRT instead of surgery is mostly due to clinico-pathological criteria, but a certain amount of patients with NSCLC might be intolerant to surgical resections due to impaired pulmonary function. Possible extensions include pulmonary function tests such as FOT, which provide information about lung capacity and tissue mechanics [36,54]. Measurements of lung function with non-invasive devices (i.e., FOT) in fragile patients with lung cancer would help to determine the functional consequences of irradiating small fields in lungs and establish evidence based criteria for eligibility for SBRT schemes on the basis of lung function. Further research should be undertaken to investigate the influence of the presence of other medical respiratory conditions (e.g., chronic obstructive pulmonary disease) on the treatment outcome.

This study is unable to encompass the entire impact of the findings from our research, certain limitations of this study being worth mentioning: the relative small sample size, given the time constraint for performing the three visits; the heterogeneity of the small group cohort that doesn’t allow a classification of the regimens; the lack of an automatic software for measuring the tumor volume in all 3 phases, requiring the physician validation.

Future work will address the development of a model prediction of acute and late toxicity after radiotherapy in lung cancer, the correlation of tumor and late toxicity with tissue heterogeneity present in the lungs, the parameters configuration of PKPD model in a bigger group of patients, the inclusion of other therapies in the treatment regimen.

## 5. Conclusions

This paper provided a feasibility analysis of a pharmacokinetic-pharmacodynamic model of tumor progression and calibration with a real tumor volume patient dataset. Our study is a proof of concept in humans that the PKPD modeling can anticipate the SBRT effect in certain groups of NSCLC patients, predict drug-drug and drug-body interactions, and guide the dose and treatment regimens. The use of such models greatly aids the support to further personalise the radiotherapy treatment in combination therapies such as immuno- and antiangiogenesis therapies. The use of such interdisciplinary patient models enables faster clinical onset relevance evaluation while allowing to work with sparse dataset information. The direct benefit is to allow working from lower numbers of patients in clinical trials, therefore shortening the time to the result availability.

## Figures and Tables

**Table 2:** Tumor and treatment related characteristics of participants completing the study.

Patients ID	Time between Tumor Measurements (Days ≈ Months)	Total Dose (Gy)	No. of Fractions	Total Duration of RT Treatment (Days)	Days of Treatment	Tumor Colume GTV before RT (cm${}^{3}$)	Tumor Volume GTV after RT (cm${}^{3}$)	Mean Lung Dose (Gy)	Total Lung Volume (cm${}^{3}$)	V5 Lungs (%)	V20 Lungs (%)
001	191 d ≈ 7 m	54	3	6	1-3-6	0.6	0.2	1.2	4870.8	6.1	0.9
002	63 d ≈ 2 m	48	4	8	1-3-5-8	0.9	0	2.7	2653.7	13	2.8
003	123 d ≈ 4 m	54	3	6	1-3-6	1	0.5	2.4	3758.1	12	1.7
005	126 d ≈ 5 m	54	3	6	1-3-6	0.4	0 *	1.1	5031.4	5	0.9
006	124 d ≈ 4 m	48	4	8	1-3-5-8	4.2	1.4	2.1	5263.3	1.6	2.1
008	109 d ≈ 4 m	48	4	8	1-3-6-8	18	1.5	3.4	3598.8	13.9	4.5
009	90 d ≈ 3 m	54	3	6	1-3-6	5.7	3.3	2.5	5485.3	12.4	3
010	169 d ≈ 6 m	48	4	9	1-3-6-9	4.3	2	1.9	3932.6	8.6	2
011	172 d ≈ 6 m	48	4	8	1-3-6-8	5.4	2.8	5.3	3340.5	34.3	4.3
012	98 d ≈ 4 m			7				7.3	2335	31.5	13
les.	1	54	3		1-4-6	1.2	1.6				
les.	2	54	3		3-5-7	0.9	0				
les.	3	54	3		3-5-7	0.9	0.3				
013	125 d ≈ 4 m			10				5	2886.4	28	4.8
les.	1	48	4		2-6-8-10	0.9	0.4				
les.	2	48	4		1-3-7-9	5.6	4.6				
014	150 d ≈ 5 m	54	3	6	1-3-6	2.6	0 *	3.7	2229	15.1	6
015	112 d ≈ 4 m	60	8	17	1-3-6-8-10-13-15-17	10.5	11	4.5	3307.4	17	6.3
016	124 d ≈ 4 m	48	4	10	1-3-7-10	20	2.8	3.7	4477	17.5	4
018	115 d ≈ 4 m	54	3	7	1-3-7	1.5	0.3	1	4463.8	4.5	1.3
019	103 d ≈ 4 m	34	1	1	1	0.3	0.2	1.2	3336.3	5.9	0.7
020	145 d ≈ 5 m			9				1.7	2602.2	7.6	1.7
les.	1	48	4		1	0.6	0.6				
les.	2	34	1		2-4-7-9	1.1	0				
021	133 d ≈ 5 m	48	4	11	1-3-8-11	0.6	0 *	1.9	2392.4	9.6	1.6
022	123 d ≈ 4 m	54	3	6	1-2-6	2.1	0 *	3.2	2830	16.5	3.4

Patients ID 004, 007 and 017 are missing from reported data due to clinical reasons. Patients ID 016 and 022 have missing data for FOT measurements, but the tumor measurements are complete. *Abbreviations*: GTV = Gross tumor volume, les = lesion number, d = days, m = months. * Tumor volume not measurable, area of radiation pneumonitis, no nodule detectable.

## Data Availability

The authors confirm that the data supporting the findings of this study are available within the article.

## Abbreviations

The following abbreviations are used in this manuscript:

BED	Biological Effective Dose
COPD	Chronic Obstructive Pulmonary Disease
CT	Computed Tomography
CTCAE	Common Terminology Criteria for Adverse Events
DVH	Dose-volume histogram
ECOG	Eastern Cooperative Oncology Group
FOT	Forced Oscillation Technique
GCP	Good Clinical Practice
GOLD	Global Initiative for COPD
GTV	Gross tumor volume
GZA	GasthuisZusters Antwerpen
ICF	Informed consent form
MLD	Mean lung dose
NA	Not applicable
NSCLC	Non-small cell lung cancer
PKPD	Pharmacokinetic-Pharmacodynamic
RP	Radiation pneumonitis
RT	Radiotherapy
SBRT	Stereotactic Body Radiation Therapy
TLV	Total lung volume
TNM	Classification of Malignant tumors
